# Ceramide Composition in Exosomes for Characterization of Glioblastoma Stem-Like Cell Phenotypes

**DOI:** 10.3389/fonc.2021.788100

**Published:** 2022-01-21

**Authors:** Raquel M. Melero-Fernandez de Mera, Alma Villaseñor, David Rojo, Josefa Carrión-Navarro, Ana Gradillas, Angel Ayuso-Sacido, Coral Barbas

**Affiliations:** ^1^ Centre for Metabolomics and Bioanalysis (CEMBIO), Department of Chemistry and Biochemistry, Facultad de Farmacia, Universidad San Pablo-CEU, CEU Universities, Madrid, Spain; ^2^ Unidad de Tumores Sólidos Infantiles, Instituto de Investigación de Enfermedades Raras (IIER), Instituto de Salud Carlos III (ISCIII), Madrid, Spain; ^3^ Centro de Investigación Biomédica en Red de Enfermedades Raras, Instituto de Salud Carlos III (CB06/07/1009; CIBERER-ISCIII), Madrid, Spain; ^4^ Institute of Applied Molecular Medicine (IMMA), Department of Basic Medical Sciences, Facultad de Medicina, Universidad San Pablo CEU, CEU Universities, Madrid, Spain; ^5^ Brain Tumor Laboratory, Faculty of Experimental Sciences and Faculty of Medicine, Universidad Francisco de Vitoria, Madrid, Spain; ^6^ Fundación Vithas, Grupo Vithas Hospitales, Madrid, Spain

**Keywords:** glioblastoma, cancer stem cells, LC-MS, exosomes, ceramides, untargeted lipidomics, proneural phenotype, mesenchymal phenotype

## Abstract

Glioblastoma (GBM) is one of the most malignant central nervous system tumor types. Comparative analysis of GBM tissues has rendered four major molecular subtypes. From them, two molecular subtypes are mainly found in their glioblastoma cancer stem-like cells (GSCs) derived *in vitro*: proneural (PN) and mesenchymal (MES) with nodular (MES-N) and semi-nodular (MES-SN) disseminations, which exhibit different metabolic, growth, and malignancy properties. Many studies suggest that cancer cells communicate between them, and the surrounding microenvironment, *via* exosomes. Identifying molecular markers that allow the specific isolation of GSC-derived exosomes is key in the development of new therapies. However, the differential exosome composition produced by main GSCs remains unknown. The aim of this study was to determine ceramide (Cer) composition, one of the critical lipids in both cells and their cell-derived exosomes, from the main three GSC phenotypes using mass spectrometry-based lipidomics. GSCs from human tissue samples and their cell-derived exosomes were measured using ultra-high-performance liquid chromatography-quadrupole time-of-flight mass spectrometry (UHPLC/Q-TOF-MS) in an untargeted analysis. Complete characterization of the ceramide profile, in both cells and cell-derived exosomes from GSC phenotypes, showed differential distributions among them. Results indicate that such differences of ceramide are chain-length dependent. Significant changes for the C16 Cer and C24:1 Cer and their ratio were observed among GSC phenotypes, being different for cells and their cell-derived exosomes.

## Introduction

Glioblastoma (GBM) is the most prevalent and malignant primary brain tumor (1). It represents 30% of all central nervous system tumors (CNSTs), and 80% of primary malignant CNSTs, having an incidence of 5 per 100,000 persons (2). Standard treatment consists of maximal surgical resection, followed by radiotherapy with or without concomitant and adjuvant temozolomide. Such treatment hardly increases patient survival and leads to a median overall survival of only 12–18 months following diagnosis (3–5). So far, clinical trials with new therapeutic approaches have shown non-significant benefits (6–9). Treatment failure is due in part to the presence of self-renewing highly tumorigenic glioblastoma cancer stem-like cells (GSCs) that contribute to tumor initiation and persevere to both chemotherapy and radiotherapy (1).

The evidence points to GSCs as a more reliable preclinical GBM model than traditional cancer cell lines, and because of that, many efforts have been made to isolate and culture GSCs in order to study their contribution to the tumorigenic processes, as well as to identify new therapeutic targets and biomarkers for diagnostics, prognostics, GBM stratification, treatment selection, and follow-up response to therapy (10, 11).

GSCs display intra- and inter-tumor heterogeneity (12, 13). These cells possess the genomic and genetic alterations found in the original tumor and thus develop similar histopathological features observed in the patients when implanted in murine models (14). GBM tissues have been divided into four different subtypes (classical, neural, proneural, and mesenchymal) based on molecular analysis (15). These molecular profiles have been partially found also in GBM tissue-derived GSCs. Comparative analysis of different GSC collections derived from GBM tissue reveals mainly two different GSC subtypes: the proneural-like phenotype (PN) and mesenchymal-like phenotype (MES) (16–19). Additionally, studies in xenograft mice models have described two different dissemination patterns from MES-derived GSC (20, 21): a nodular pattern (MES-N) with well-defined boundaries and a semi-nodular pattern (MES-SN) with a visible nodular-like tumor mass but not well-defined nodular boundaries. The PN phenotype has a longer survival rate (20, 22). Although aggressive treatment, in GSC-based xenograft mice models, with conventional chemotherapeutics significantly reduces mortality in both MES phenotypes, it does not alter survival in the PN phenotype. This resistance from the PN phenotype has also been observed in several therapies such as anti-angiogenic treatment. *In vitro* assays have shown that the PN phenotype is more sensitive to microtubule-stabilizing drugs such as paclitaxel (which is also sensitive for the MES-N phenotype but not MES-SN) (20). Profiling-based classification may therefore have the highest clinical relevance for suggesting different therapeutic strategies, as there is no existing diagnostic test between these phenotypes.

Several cancer tumors shed materials into the peripheral circulation such as circulating tumor cells (CTCs) and soluble proteins, which are recently exploited as surrogate markers of tumor staging and response to therapy (23–25). However, in the case of the central nervous system, which lies behind a partially intact blood–brain barrier, this does not release CTCs and thus is not commonly associated with detectable soluble protein biomarkers. In this case, glioma cells are able to release extracellular vesicles (EVs), including exosomes and microvesicles (MVs), which cross the blood–brain barrier and could be detected within blood (26). The EVs produced by GSCs could offer a potential new way for detection and treatment monitoring.

Many studies suggest that cancer cells communicate with each other and their neighboring cells *via* exosomes (27). These are small (30–150 nm diameter) double-membrane-bound vesicles (28). Exosomes interact with the receptor ligand and are internalized or fused with the target cell membrane to send their content into their cytosol, altering the physiological state of the recipient cell (29). They are considered as signal packets containing proteins, mRNA, miRNA, transcription factors, lipids, and other active constituents (30, 31) and have the pleiotropic capacity in regulating tumor growth and progression (32). Particularly, changes of lipid metabolism mediated by exosomes are increasingly recognized as a characteristic of cancer cells and may be a contributing factor to tumor progression and metastatic behavior (33, 34). Thus, exosomes have been suggested as diagnosis, prognosis, and therapeutic biomarkers of different diseases (29, 34).

Regarding the lipid composition in exosomes, it is well-known that specific lipid classes are enriched in exosomes compared to their parent cells, thus suggesting a specific role of lipid composition in exosome formation, secretion, and function (34, 35). Lipids not only have a structural role in the exosome membrane but also are key players in their formation and signaling in both the producer and recipient cells (35). However, only a few studies have investigated the function of lipids in exosomes, and more specifically ceramides, one of the critical lipids that are enriched in this type of EVs (34, 36, 37).

Ceramides (Cer) are the central core of sphingolipid metabolism, as they are key players in intracellular signaling and are involved in apoptosis, cell senescence, proliferation, cell growth, and differentiation (38). Different studies have demonstrated that ceramide production and its biological role are linked to the length of the fatty acyl chain (39). As an example, levels of C16 Cer, C18 Cer, C24 Cer, and C24:1 Cer were observed significantly altered on tumor dignity in human breast cancer, human head and neck squamous cell carcinomas (HNSCCs), and colon cancer (39–41). Data attributed to cancer-specific intercellular transfer molecules to exosomes are still limited. In-depth characterization of ceramides contained in exosomes will help to elucidate their precise biological functions in GBM. To this end, lipidomics is the science advocated for their analysis (42–44). Currently, liquid chromatography coupled to mass spectrometry (LC-MS) has become one of the most powerful techniques for the analysis of lipids owing to its high sensitivity, specificity, and high-throughput capabilities (45, 46).

The aim of this study was to evaluate the MS-based lipidomics for the characterization of ceramide species in both the exosomes and their parent cells from the three main GSC phenotypes: PN, MES-N, and MES-SN. Structural characterization provided 14 ceramide species from cells and exosomes. We observed that the distribution of ceramides between the parent cells and their cell-derived exosomes is different for all three phenotypes analyzed. Significant changes for the C16 Cer and C24:1 Cer were observed between phenotypes, being also different for cells and their cell-derived exosomes.

Our LC/MS-based lipidomic analysis provides a platform for comprehensive lipidome profiling, focusing in particular on ceramides species, across GSCs and their derived exosomes, that will facilitate subsequent functional studies aimed at elucidating the role of specific cellular or exosome ceramides in the onset and progression of glioblastoma cancer, or to identify specific ceramides that could serve as potential effective diagnostic or prognostic disease biomarkers.

## Results

### Metabolic Profile of GSC Phenotypes From Cells and Exosomes

**Figure 1:**
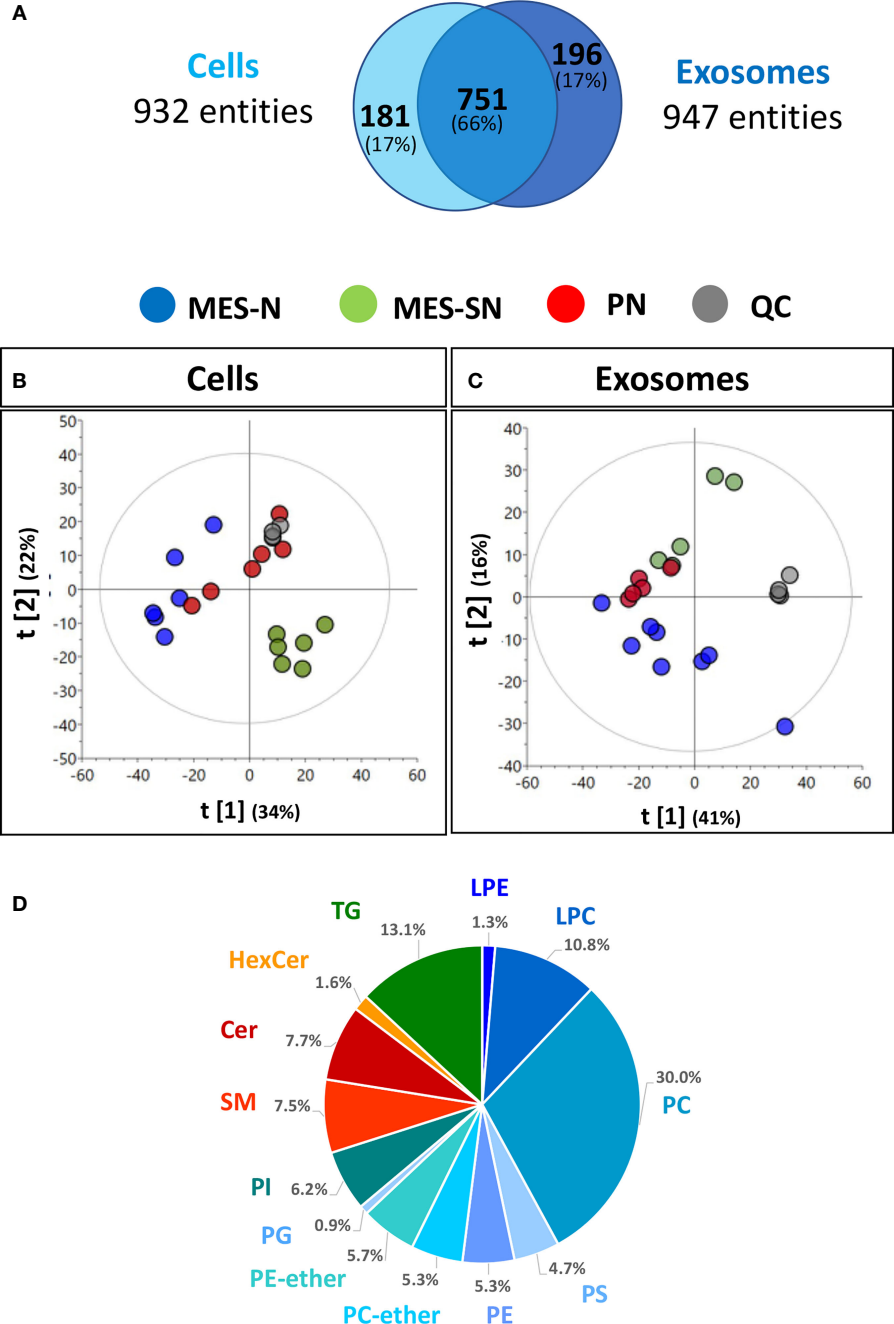
Metabolic profile description of GSC phenotypes from cells and their derived exosomes. **(A)** Chemical entities in cells and exosomes that passed the quality filters, **(B, C)** Principal component analysis (PCA) models of cells and exosomes: quality control injections (QC, • dark-gray dot), MES-N (•; blue dot), MES-SN (•; green dot), and PN (•; red dot). Unit variance scaling was used for both models. **(D)** Pie chart showing the percentages of the number of lipid classes for cells. LPE, lysophosphatidylethanolamines; LPC, lysophosphatidylcholines; PC, phosphatidylcholines; PS, phosphatidylserines; PE, phosphatidylethanolamines; PC-ether, ether-phosphatidylcholines; PE-ether, ether-phosphatidylethanolamines; PG, phosphatidylglycerols; PI, phosphatidylinositols; SM, sphingomyelins; Cer, ceramides; HexCer, hexosylceramides; TG, triacylglycerols.

To obtain the ceramide profile, up to eight replicates of the main GSC phenotypes (PN, MES-N, MES-SN) and their exosomes were analyzed using untargeted ultra-high-performance liquid chromatography-quadrupole time-of-flight mass spectrometry (UHPLC/Q-TOF-MS) based lipidomics. After data treatment, 932 and 947 chemical entities in each sample were obtained for cells and exosomes, respectively, using positive electrospray ionization (ESI) mode. These chemical entities passed the different quality filters [blank subtraction, presence in quality control samples (QCs; >50%) and sample groups (>75%), and coefficient of variation in QCs (<20%)] for each matrix (cells and exosomes). The shared entities between cells and exosomes were approximately 66% (**Figure 1A**).

To assess the correct performance of the LC-MS technique and the quality of the acquired data, cells and exosome data were projected independently on a principal component analysis (PCA) model (**Figures 1B, C**). Clustering of the QC injections in the non-supervised PCA plots for cells and exosomes indicated the high quality of the data and showed high reproducibility, while the dispersion of the samples showed the biological variability of the three phenotypes. In the case of cells, the separation by the first component [t[1)], the MES-N phenotype seemed to be opposite to their counterpart MES-SN, being the PN phenotype between both MES-based phenotypes (**Figure 1B**). Regarding the exosomes, the same trend was observed as in cells, except for being separated by the second component [t[2)] (**Figure 1C**). As we observed clustering of the QC samples and each phenotype group for cells and exosomes, we used both data matrices to look for ceramide composition.

In order to identify the number of lipid species globally, a pool of cell samples was analyzed using the iterative method. Lipid classes are depicted in a pie chart using the Agilent Lipid Annotator software tool (version 1.0) (**Figure 1D**). In total, 13 lipid classes were observed merging both positive and negative electrospray ionization (ESI+ and ESI−) being the sphingolipids containing ceramides the 16.8% of the total number content.

### Structural Characterization of Ceramides

Using UHPLC/Q-TOF-MS as analytical tool, a total of 14 Cer species ranging from 14 to 26 carbons have been separated and readily detected in this study. Data from tandem mass spectrometry (ESI-MS/MS), in both ESI positive (+) and ESI negative (−) ionization modes, have yielded multiple sets of fragment ions, regarding their sphingoid backbone and fatty acyl substituents, which have been deeply analyzed and have led to confident structure assignment as shown in **Table 1**. All fragmentations observed have been confirmed based on previously published results and predictions (45, 47, 48).

**Table 1:** Complete characterization of ceramides in cells and exosomes from GCS phenotypes using UHPLC/ESI(+)-Q-TOF-MS/MS.

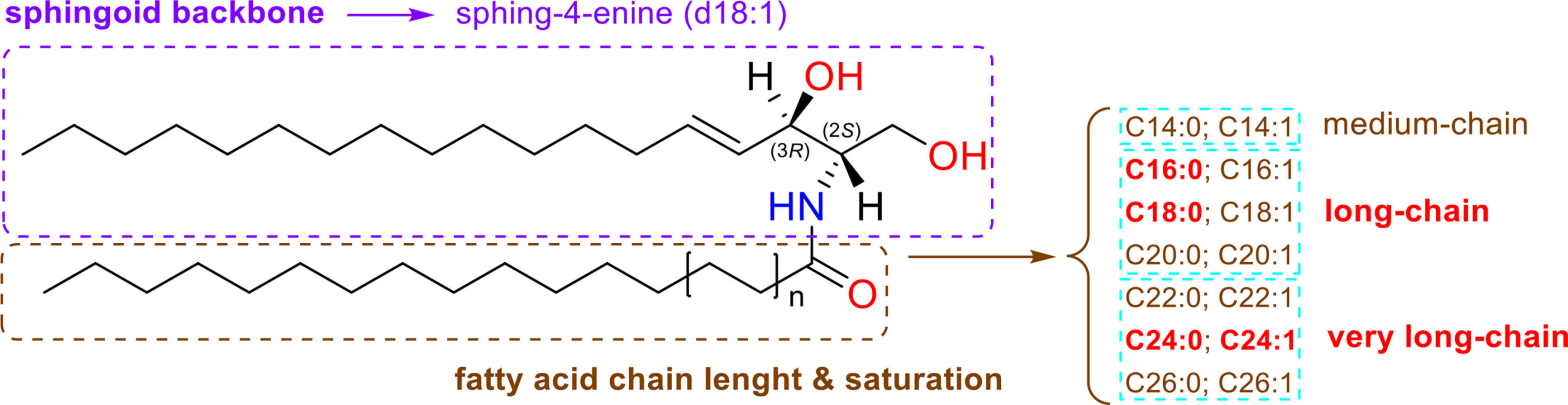							LCB-related ions—common ions for (dLCB d18:1)^a,b^			
							[LCB+H-FA-H_2_O]^+^ = 282.2788			
							[LCB+H-FA-2H_2_O]^+^ = 264.2688			
							[LCB+H-FA-HCOH-H_2_O]^+^ = 252.2688			
							[LCB+H-FA-2H_2_O-NH_3_]^+^ = 247.2426			
Ceramide			Ceramide common ions				FA-related ions		Shorthand notations	
Cer	Rt(min)	Monoisotopic mass	[M+H]^+^	[M+H-H_2_O]^+^	[M+H-2H_2_O]^+^	[M+H-H_2_O-HCHO]^+^	[M+H-282]^+^	Type	Abbrev chains	Abbrev
**C14 Cer**	11.2	509.4808	510.4881	492.4770	474.4660	462.4674	228.2310	**C14:0**	Cer 18:1;O2/14:0	Cer 32:1;O2
**C14:1 Cer**	10.3	507.4594	508.4724	490.4615	472.4515	460.4511	226.3829	**C14:1**	Cer 18:1;O2/14:1	Cer 32:2;O2
**C16 Cer**	13.1	537.5121	538.5194	520.5080	502.4980	490.4941	256.2631	**C16:0**	Cer 18:1;O2/16:0	Cer 34:1;O2
**C16:1 Cer**	12.3	535.4962	536.5037	518.4932	500.4820	488.4812	254.2476	**C16:1**	Cer 18:1;O2/16:1	Cer 34:2;O2
**C18 Cer**	14.8	565.5434	566.5500	548.5420	530.5288	518.5290	284.2930	**C18:0**	Cer 18:1;O2/18:0	Cer 36:1;O2
**C18:1 Cer**	13.7	563.5277	564.5342	546.5239	528.5133	516.5133	282.4911	**C18:1**	Cer 18:1;O2/18:1	Cer 36:2;O2
**C20 Cer**	16.5	593.5747	594.5820	576.5701	558.5609	546.5640	312.3260	**C20:0**	Cer 18:1;O2/20:0	Cer 38:1;O2
**C20:1 Cer**	15.4	591.5521	592.5663	574.5549	556.5449	544.5448	310.3100	**C20:1**	Cer 18:1;O2/20:1	Cer 38:2;O2
**C22 Cer**	18.1	621.6060	622.6147	604.6017	588.6061	574.5871	340.3575	**C22:0**	Cer 18:1;O2/22:0	Cer 40:1;O2
**C22:1 Cer**	17.5	619.5879	620.5976	602.5859	586.6014	572.5751	338.3416	**C22:1**	Cer 18:1;O2/22:1	Cer 40:2;O2
**C24 Cer**	19.4	649.6373	650.6444	632.6336	614.6191	602.6263	368.3850	**C24:0**	Cer 18:1;O2/24:0	Cer 42:1;O2
**C24:1 Cer**	18.3	647.6216	648.6298	630.6183	612.6063	600.6064	366.3733	**C24:1**	Cer 18:1;O2/24:1	Cer 42:2;O2
**C26:1 Cer**	19.6	675.6529	676.6591	658.6489	656.6722	628.6390	394.4039	**C26:1**	Cer 18:1;O2/26:1	Cer 44:2;O2

a The **m/z** value for the base peak are given in bold type.

b General structure of ceramides (N-acyl-D-erythro-(2S,3R)-sphingosines): Ceramides consist of a long-chain aliphatic amino alcohol that have a trans-double bond at the C-4-5 position that is referred to the long-chain base (LCB). The sphing-4-enine LCB is designated as d18:1, which is attached via an amide linkage at C-2 to a fatty acyl chain (FA), varied by the chain length. Naturally occurring ceramide exists in the D-erythro conformation (2S,3R). Common names for the most significant saturated fatty acid C16:0, C18:0, and C24:0 are palmitic acid, stearic acid, and lignoceric acid, respectively. The common name for the most significant monounsaturated fatty acid C24:1 is nervonic acid.

The nomenclature used for the annotation of the fragment ions observed, as well as the mechanisms leading to their formation, are based on general literature recommendations (48, 49). Briefly, the designation of Cer is in the form of dLCB/FA, with d denoting a dihydroxy long-chain base (LCB), namely, sphing-4-enine, which is designated as d18:1, and FA designating a non-hydroxylated fatty acid (**Table 1**). The observation of specific fragment ions reflecting the dLCB and FA substituents is essential in the structural identification of ceramides that often consist of many isomers.

### Formation of Positive Ions and General MS/MS Fragmentation Characteristics of [M+H]^+^

**Figure 2:**
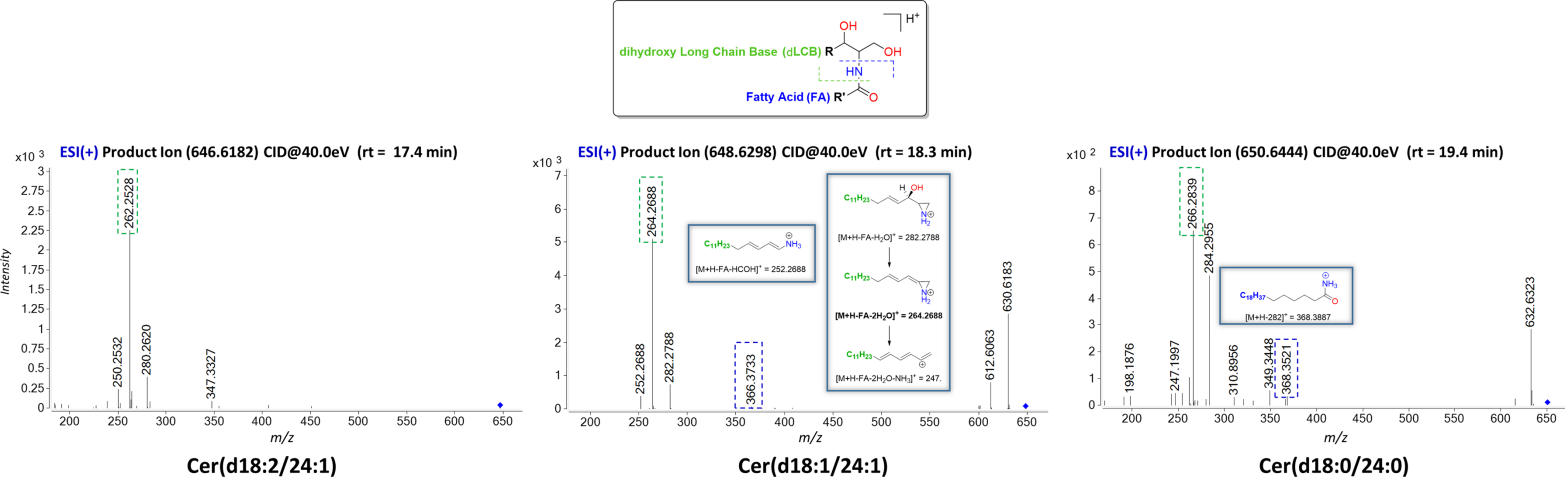
Characteristic ESI(+)-MS/MS fragmentation pattern for structural characterization of the dLCB composition of the three types of sphingoid backbone (d18:2, d18:1, and d18:0). The fragments ions arising from losses of H_2_O are common ions seen for all ceramides. Fragment ions possessing dLCB are framed in a green dashed square, and fragment ions possessing an FA chain structure are framed in a blue dashed square. Proposed structures for main ion fragments observed based on published predictions (47).

Cer produce predominately protonated species under ESI+ mode such as [M+H]^+^ and [M+Na]^+^ followed by further loss of H_2_O (18.012 Da). The MS/MS spectra contain abundant specific fragment ions that identify the dLCB of the molecules, which allows differentiation among different ceramide subclasses (**Table 1**). Thus, characteristic ions specific to the sphingoid backbone are decisive for the structural elucidation of ceramides, e.g., *m*/*z* 266.2839, *m*/*z* 264.2688, and *m*/*z* 262.2528 ions for the assignment of sphingadienine (d18:2), sphing-4-enine (d18:1), and sphinganine (d18:0) backbones, respectively (45) (**Figure 2**).

### Formation of Negative Ions and General MS/MS Fragmentation Characteristics of [M-HCOOH-H]^−^

**Figure 3:**
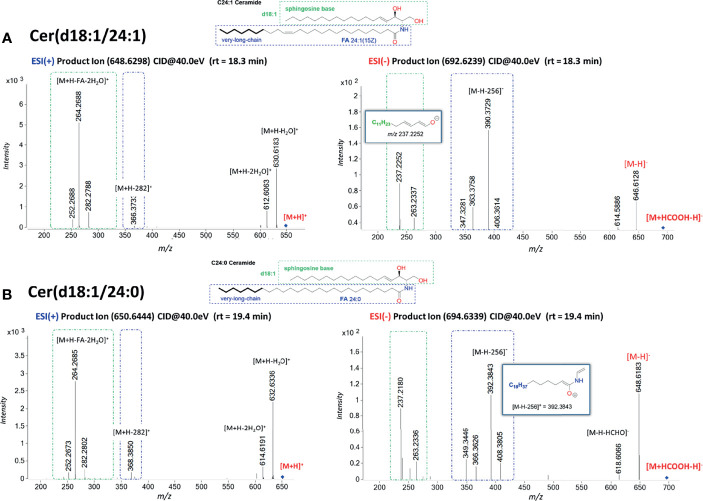
Characteristic MS/MS fragmentation pattern for structural characterization of fatty acyl composition. **(A)** MS/MS spectra in ESI(+) and ESI(−) for Cer(d18:1/24:1); **(B)** MS/MS spectra in ESI(+) and ESI(−) for Cer(d18:1/24:0). The fragment ions arising from losses of H_2_O, HCHO, and H_2_O + CHOH are common ions seen for all Cer. Fragment ions possessing dLCB are framed in a green dashed square, and fragment ions possessing an FA chain structure are framed in a blue dashed square. Cer with saturated FA are more retained in reverse-phase chromatography than the one with unsaturated double bonds. The fragment ion of [M-H-256]^−^ arising from the combined losses of H_2_O and the LCB as an aldehyde is a common ion observed for ceramides with a dLCB(18:1) (47, 49).

Ceramides produce predominately adducts with formate ion such as [M+HCOOH-H]^−^ when subjected to ESI in negative ionization mode followed by further loss of HCOOH (46.0111 Da) and of HCHO (30.0123 Da). Predominant ions from the MS/MS spectra are shown in **Table 2**. Complete structural characterization of ceramides containing saturated and unsaturated fatty acyl substituent is exemplified by Cer(d18:1/24:0) and Cer(d18:1/24:1) at *m*/*z* 692.6239 and *m*/*z* 694.6339, respectively, which yielded the MS/MS spectra shown in **Figures 3A, B**. Both spectra also contained the predominant ions of *m*/*z* 406.3614 and *m*/*z* 390.3729 and ions of *m*/*z* 363.3758 and *m*/*z* 347.3281 that identified the 24:1 FA chain, along with the ions of *m*/*z* 263.2337 and *m*/*z* 237.2252 that recognized d18:1 as LCB, giving the assignment of the C24:1 Cer structure (48).

**Table 2:** Characterization of ceramides present in cells and exosomes from GCS phenotypes using UHPLC/ESI(−)-Q-TOF-MS/MS^a^.

LCB-related ions—common ions for (dLCB d18:1): *m*/*z* 263.2378 and *m*/*z* 237.2232							
Ceramide			Ceramide common ions^b^		FA most significant related ions^b^		Shorthand notations
Cer	Rt(min)	Monoisotopic mass	[M+HCOOH-H]^−^	[M-H]^−^	[M-H-256]^−^	Type	Abbrev chains
**C14 Cer**	11.2	509.4808	554.4781	508.4730	**252.2333**	**C14:0**	Cer 18:1;O2/14:0
**C14:1 Cer**	10.3	507.4594	552.4631	506.4573	**250.2132**	**C14:1**	Cer 18:1;O2/14:1
**C16 Cer**	13.1	537.5121	582.5106	536.5044	**280.2646**	**C16:0**	Cer 18:1;O2/16:0
**C16:1 Cer**	12.3	535.4962	534.4894	534.4888	**278.2493**	**C16:1**	Cer 18:1;O2/16:1
**C18 Cer**	14.8	565.5434	610.5400	564.5358	**308.2960**	**C18:0**	Cer 18:1;O2/18:0
**C18:1 Cer**	13.7	563.5277	608.5254	562.5200	**306.2800**	**C18:1**	Cer 18:1;O2/18:1
**C20 Cer**	16.5	593.5747	638.5709	592.5670	**336.3275**	**C20:0**	Cer 18:1;O2/20:0
**C20:1 Cer**	15.4	591.5521	636.5570	590.5515	**334.3111**	**C20:1**	Cer 18:1;O2/20:1
**C22 Cer**	18.1	621.6060	666.6025	620.5983	**364.3579**	**C22:0**	Cer 18:1;O2/22:0
**C22:1 Cer**	17.5	619.5879	664.5866	618.5835	**362.3432**	**C22:1**	Cer 18:1;O2/22:1
**C24 Cer**	19.4	649.6373	694.6339	648.6306	**392.3898**	**C24:0**	Cer 18:1;O2/24:0
**C24:1 Cer**	18.3	647.6216	692.6199	646.6128	**390.3736**	**C24:1**	Cer 18:1;O2/24:1
**C26:1 Cer**	19.6	675.6529	720.6487	674.64563	**418.4050**	**C26:1**	Cer 18:1;O2/26:1

a The m/z value for the base peak are given in bold type.

b For complete characterization and details, see reference (48).

The difference in chromatographic retention for C24 Cer as compared to C24:1 Cer is explained by the polarity difference within 1 min of their measured retention time.

### Ceramide Profile in Cells and Exosomes

**Figure 4:**
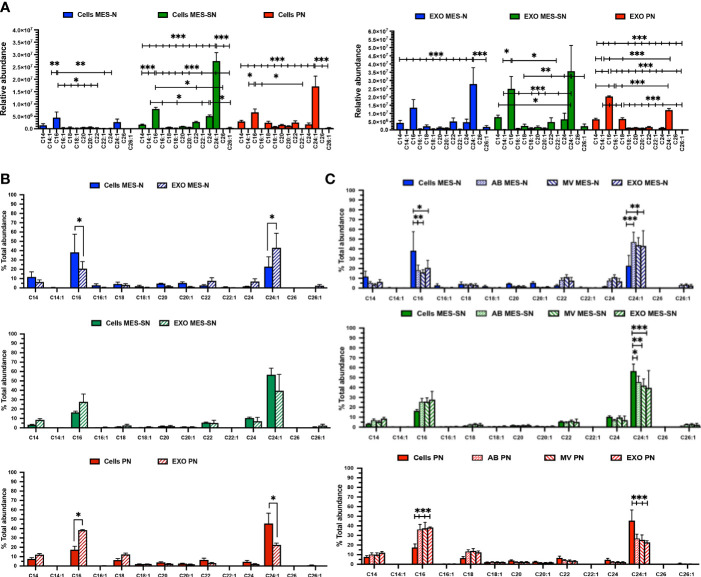
Ceramide profile in cells, exosomes (EXO), MV, and AB for the three GBM phenotypes. **(A)** Ceramide profile for the three phenotypes for cells and their derived exosomes (EXO). **(B)** Ceramide distribution between exosomes and their parent cells for each phenotype independently. **(C)** Ceramide distribution among MV, AB, exosomes, and their parent cells for each phenotype independently. In blue, MES-N phenotype; in green, MES-SN phenotype; and in red, PN phenotype. For all bar graphs, error bars represent the mean ± SEM. *p < 0.05; **p < 0.01; and ***p < 0.001.

The ceramide profile characterized in previous sections was searched into the experimental samples for cells and exosomes in the three phenotypes. The first aim was to compare the abundance of the ceramides inside each phenotype for cells and exosomes independently (**Figure 4A**). For both, cells and exosomes; C16 Cer and C24:1 Cer were the most abundant ceramides for the three phenotypes. Additionally, C18 Cer, which has been reported as one of the most abundant ceramides in the brain comprising 69% of the total ceramide profile (50), was not the highest in the three GSC phenotypes for cells and exosomes as we observed in this study.

Moving forward, to compare the distribution of ceramide species in exosomes and their parent cells, ceramides were normalized to the total abundance of the profile expressed in percentage (%) independently for each phenotype (**Figure 4B**). Regarding the phenotype MES-SN, a significant increase of the C24:1 Cer in exosomes compared to their cells was observed. In the case of MES-N, no significant differences in distribution for ceramides were observed between the exosomes and their parent cells. However, for the PN phenotype, a significant increase of C16 Cer and a significant decrease of C24:1 Cer in exosomes compared to their parent cells were observed. This trend is the same as MES-N but opposite to the one observed for the MES-SN phenotype. Interestingly, C14:1 Cer and C22:1 Cer were the lowest ceramides observed for exosomes and cells, and C26 Cer was not detected in cells nor in exosomes.

Looking at these previous results, ceramide distribution was searched between parent cells and other EVs isolated (see *Materials and Methods*) apart from exosomes (**Figure 4C**). The results showed that ceramides from MV and apoptotic bodies (AB) follow the same pattern as exosomes. As analysis of lipids in exosomes has been suggested as a useful approach when looking for biomarkers, exosomes were selected for further comparisons (34). Zoomed graphs of the ceramide profiles in abundance and distribution can be observed in **Supplementary Figure 1**.

To sum up this part, the MES-SN phenotype in cells showed lower levels of ceramides compared to the other phenotypes in cells. The distribution between exosomes and their parent cells showed opposite distribution for MES-SN and MES-N phenotypes.

**Figure 5:**
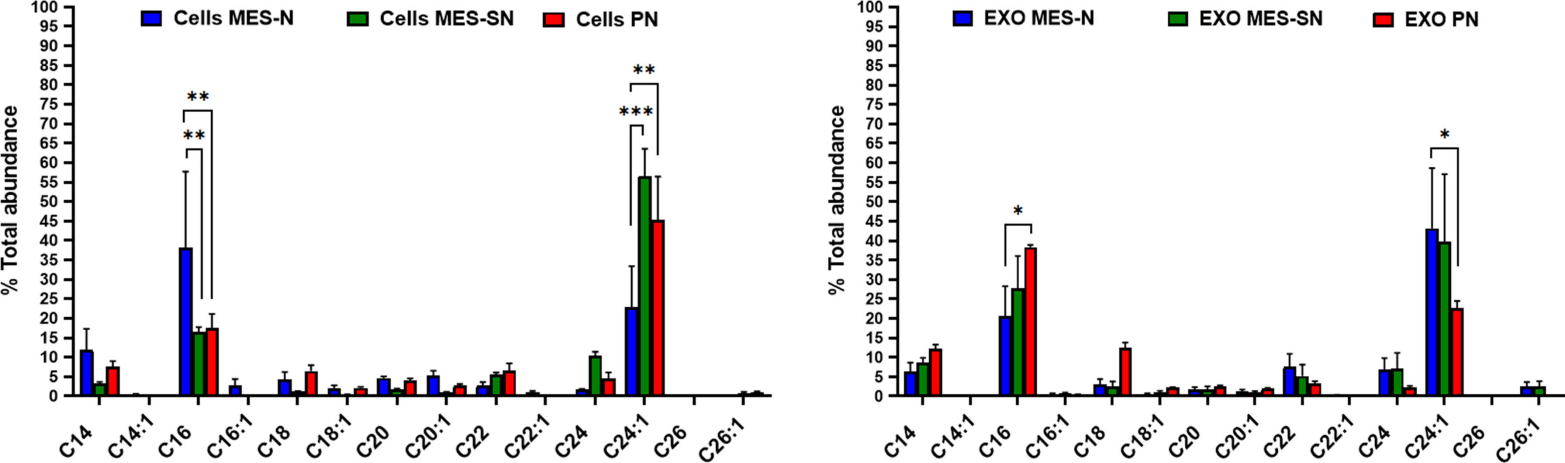
Normalized ceramide profiles among the three phenotypes in cells and exosomes. In blue, MES-N phenotype; in green, MES-SN phenotype; and in red, PN phenotype. For all bar graphs, error bars represent mean ± SEM. *p < 0.05; **p < 0.01; and ***p < 0.001.

Finally, the normalized ceramides to the total abundance in % were compared for each ceramide species among the three phenotypes inside cells and exosomes (**Figure 5**). Our LC-MS/MS analysis showed that the major ceramides species from cells and cell-derived exosomes were C16 Cer and C24:1 Cer. Previous studies have shown that levels of C16 Cer play a decisive role in apoptosis while C24:1 Cer plays one in cell proliferation. For example, in a recent study, sphingolipid profiling of human oligodendroglioma (HOG) cell-derived exosomes revealed differences in the sphingolipid composition, in particular of C16-, C24-, and C24:1-Cer species, between exosomes released constitutively or under stimulation with inflammatory cytokines, as mediators of cell death signaling (51, 52).

Recently, the ceramide species ratios were incorporated by the Mayo Clinic as an alternative option to single ceramide values (53); their use is becoming common and being implemented in the clinic (54–56). In this sense, for cardiovascular (CV) death in individuals with coronary artery disease, the ratios comparing the relative proportion of long-chain ceramides (carbon chain length 14–18) in relation to very long (carbon chain length ≥ 20) (e.g., C16/C24 Cer, C18/C24 Cer, and C24:1/C24 Cer) were observed to be significant predictors of the disease. Others (e.g., C16/C22 Cer and C16/C24 Cer) are associated with the risk of major CV events in healthy individuals or cancer mortality (55, 57, 58).

**Figure 6:**
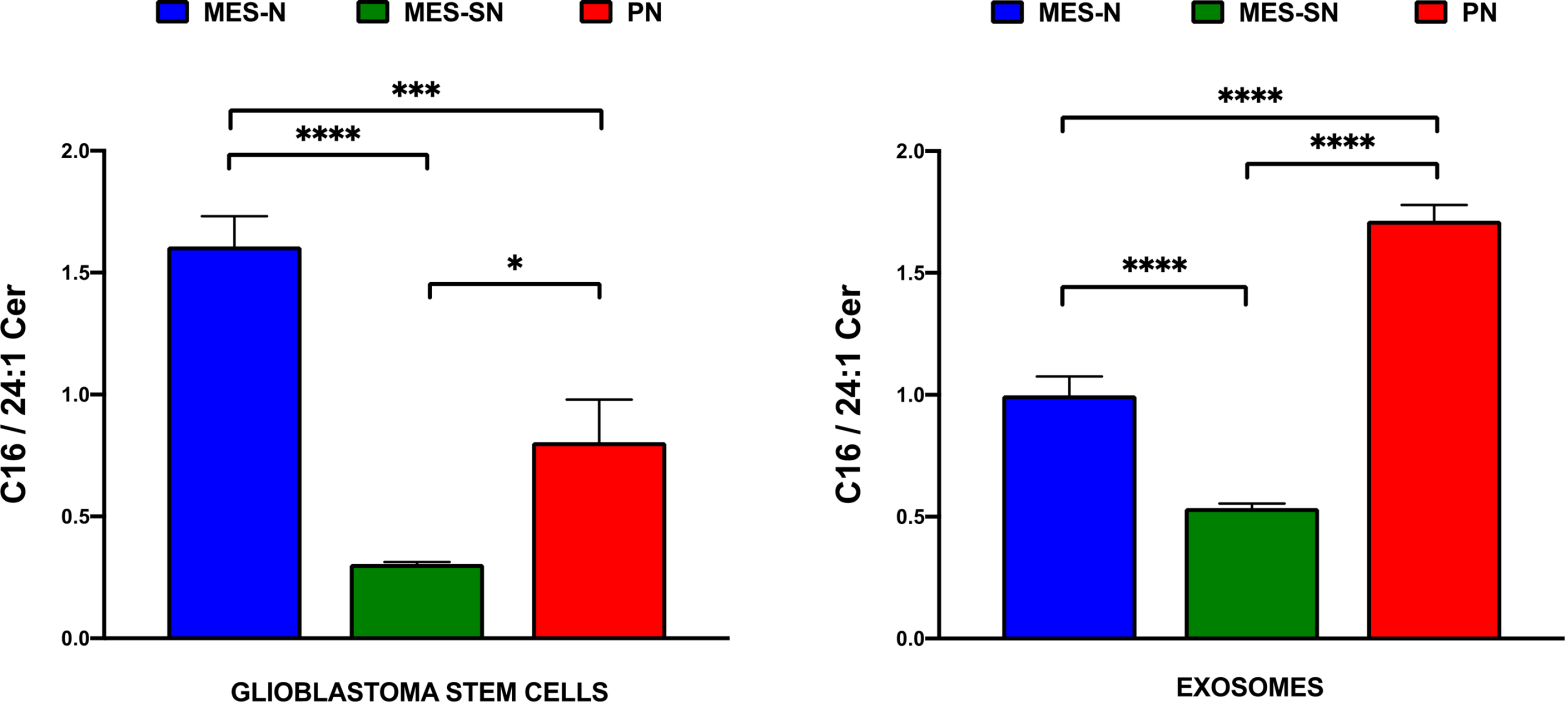
C16/C24:1 Cer ratio in GSCs and their cell-derived exosomes. In blue, MES-SN phenotype; in green, MES-N phenotype; and in red, PN phenotype. For all bar graphs, error bars represent mean ± SEM. *p < 0.05; ***p < 0.001; and ****p < 0.0001.

Following a similar approach as the Mayo Clinic, we focused on the two major species that constitute the main changes observed in our study (**Figure 5**) and which are consistent with previous works of glioma-related research (52, 54). Thus, instead of looking at the trend of each significant individual ceramide, we have found more interesting to determine the relative proportion of C16 Cer in relation to C24:1 Cer in form of a ratio in order to better express a common pattern of the changes observed (**Figure 6**). A disequilibrium between these ceramide species could be crucial for cancer progression (51, 59), and this ratio could state the balance between C16 Cer and C24:1 Cer as an important indicator to understand the behavior of the three GSC phenotypes in cells and exosomes.

As can be observed from **Figure 6** and **Table 3**, the ratio of C16/C24:1 Cer is significant among the three groups in GSCs and their derived exosomes. Regarding the cells, MES-N is the phenotype with the highest amount of C16 Cer in association with its decrease of C24:1 Cer. However, this is not the case for exosomes, where the PN phenotype showed the highest C16/C24:1 Cer ratio. For both, GSCs and their derived exosomes, MES-SN was the phenotype with the lowest values of the C16/C24:1 Cer ratio in comparison with the other two phenotypes. In addition, the comparison of the trend between cells and exosomes showed that the C16/C24:1 Cer ratio in MES-N was lower in exosomes compared to that in their progenitor GSCs. Interestingly, the MES-SN and PN phenotypes had an opposite trend where the ratio was higher in exosomes compared to that in their progenitor GSCs. These results are of great interest as they can differentiate among the three GSC phenotypes.

**Table 3:** Ceramide ratios for cells and exosome phenotypes.

Cells—ceramide ratio	MES-N		MES-SN		PN	
	Mean	SD	Mean	SD	Mean	SD
C16/C24:1 Cer	1.608	0.302	0.304	0.021	0.805	0.429
**EXO—ceramide ratio**	**MES-N**		**MES-SN**		**PN**	
	**Mean**	**SD**	**Mean**	**SD**	**Mean**	**SD**
C16/C24:1 Cer	0.998	0.174	0.535	0.053	1.714	0.132

## Discussion

GBM is the most common and aggressive brain tumor in adults (1). The prognosis of GBM patients remains poor due to their heterogeneity. The subpopulation of GSCs is primarily responsible for radiation and chemotherapy resistance and, consequently, poor patient survival (10, 11). Therefore, new therapeutic approaches are urgently needed, which require a deeper understanding of the GSCs biology, as well as identifying new molecular markers for diagnosis, response to therapy prediction, and follow-up treatments (60).

Sphingolipids are integral structural components of cell membranes that also act as critical bioactive signaling molecules to determine many aspects of cell fate and function, making them attractive research targets. Central to the sphingolipid pathway are ceramides, which are generated from the activity of six ceramide synthases (CerS1–6). These CerS are distributed differently among tissues and cell types and which selectively generate ceramides with different *N*-linked acyl chain lengths, which affects their cellular functions (61). We are just beginning to understand the biological function of these six CerS and defining the precise activities of the distinct ceramide molecular species. However, according to the literature, they seem to play a role in cancer. Many studies suggest that dysregulation of CerS contributes to the onset or progression of a variety of critical processes such as cell growth and death, autophagy, migration and invasion, angiogenesis, and inflammation (62). As such, CerS have been established to play a role in cancer progression and chemoresistance (63).

The literature indicates that ceramides are associated with important roles in apoptosis and proliferation, particularly in relation to the length of their side chain (51, 53). Previous works have established that long-chain ceramides (C16 Cer, C18 Cer, C20 Cer) are commonly associated with apoptosis, while very long-chain ceramides (C24 Cer and C24:1 Cer) are involved in cell proliferation (53). Thus, a disequilibrium among them is crucial for cancer progression (40, 60). In previous studies, the C18 Cer levels were reported significantly decreased in HNSCC tissues and glioma patient samples as compared to those in normal tissues (64, 65). Specifically, a study in GBM showed that the levels of C18 Cer were reduced up to 70% compared to those in normal tissue (66). Thus, C18 Cer and its CerS (CerS1) have been proposed as markers of tumoral suppression in clinical studies (67). In our study, C18 Cer in cells and their derived-cells exosomes was not part of the most abundant ceramides of the profile (**Figure 5**). This aspect is in line with the abovementioned that C18 Cer was found decreased in GBM tissue. Furthermore, looking at the comparison between the three GSC phenotypes used in the present work, although not significant, the lowest levels of C18 were observed in the MES-N phenotype in comparison with MES-SN and PN phenotypes in GSCs. Complementarily, in exosomes, C18 Cer was lower in MES phenotypes (MES-N and MES-SN) compared to the PN phenotype. Therefore, new experiments will be needed to find out whether this trend of C18 Cer observed in this work is actually a specific behavior of MES phenotypes compared to PN in exosomes.

Moreover, recent findings related to carcinoma cells derived from human breast, colon tumors, and HNSCC have shown higher levels of C16 Cer, C24, and C24:1 Cer compared to controls. While C16 Cer was reported in HNSCC to be anti-apoptotic (51), in other cellular models from human breast and colon tumors, C16 Cer is pro-apoptotic (68, 69). Levels of very long-chain ceramides (C24 and C24:1 Cer) were always upregulated in all the studies and according to literature have been related to the promotion of proliferation. Indeed, it was published that CerS2 overexpression, which generated very long-chain ceramides (C22-C24 Cer), offers partial protection from irradiation-induced apoptosis (68). Moreover, in a study by Wit et al. (70), an increase of C16 Cer along with a decrease of C24:1 Cer indicates a shift to a more pro-apoptotic environment in brain tissue. Other studies in ovarian and colorectal cancer have found significantly altered levels of ceramides, mainly C16 Cer, C18 Cer, C24 Cer, and C24:1 Cer, using serum and plasma samples (71–74). Regarding our results, GSCs and their cell-derived exosomes ceramides followed different patterns for each GSC phenotype (**Figures 4**, **5**).

Even though our study has been carried out in GSCs and their cell-derived exosomes and did not include control or healthy samples, our results are in accordance with those reported in tissue from colorectal tumors and breast cancer. In a study of colorectal tumor tissues, the levels of C16 Cer, C24 Cer, and C24:1 Cer were significantly elevated while levels of C18 Cer and C20 Cer showed opposite trends compared to non-tumor tissues (75). Similar results were found in breast cancer tissue regarding C16 Cer, C24:1 Cer, and C24 Cer compared with benign and normal tissue (39).

Regarding the ratio of C16/C24:1 Cer, the results are of great interest as they can differentiate among the three GSC phenotypes. However, further studies are needed to decipher the potential role of C16 Cer and C24:1 Cer in GSCs and their derived exosomes.

Some limitations need to be considered for the data presented in this work. Our study aims were to compare the ceramide distribution in the three stem cellular phenotypes of GSCs and as such in their cell-derived exosomes. However, to fully address the causality of ceramides in GSC phenotypes, a new cohort of GSC or blood samples from GBM patients is needed. Moreover, functional studies should be performed to assert the potential role of the ceramides present in GSCs and their cell-derived exosomes.

Finally, to our knowledge, this is the first study that has been carried out using GSCs and their cell-derived exosomes from patients with GBM concerning a complete characterization, composition, and distribution of ceramide profile using MS-based lipidomics.

The importance of detecting these GSC phenotypes lies in the different therapeutic approaches that may be required for each of them. From the ceramide species, C16 and C24:1 Cer seem to play an important role in GSCs and their cell-derived exosomes. According to the literature, these results might suggest that ceramides, particularly in terms of their chain length-specific effects, can be closely associated with apoptosis and cancer progression processes. However, further studies are needed in a new cohort of human GBM samples along with functional assays in these cellular models to assess their pathophysiological role in this disease. This is the first time that the characterization of ceramides in Q-TOF-MS, in both ESI(+) and ESI(−) modes, has been shown. In-depth understanding of the balance between long and very long ceramides may provide useful avenues for further research. Because sphingolipid metabolism is interconnected (e.g., ceramides can be metabolized to complex sphingolipids or degraded to sphingosine), further studies integrating other sphingolipid classes such as sphingomyelin and hexosyl ceramides are also needed.

## Material and Methods

### GCSs Isolation and Culture

Tumor cells with stem cell-like properties can be cultured from human glioblastomas by using conditions that select for the expansion of neural stem cells (76). GSCs were isolated from GBM surgical samples diagnosed by the pathologist of the Hospital Universitario La Fe and cultured according to the protocol described previously (20, 77). Briefly, the surgical samples were collected at room temperature in normal saline solution and transferred to the stem cell laboratory in Hank’s balanced salt solution (Invitrogen Corp., Carlsbad, CA, USA). Dissociated cells were plated and incubated in M21 media (Invitrogen Corp.) supplemented with basic fibroblast growth factor (FGF) (20 ng/ml), epidermal growth factor (EGF; 20 mg/ml), and heparin (2 μg/ml). Cultures were serially passaged every 15 to 20 days by dissociation followed by replating into fresh growth medium for periods as long as 20 months. This technique permits the isolation of a uniform population of cells. Self-renewal and immunocytochemical experiments proved the feasibility of long-term expansion of a slowly dividing capable of generating new neurons and astrocytes. These cells generated a large amount of progeny and possessed significant self-renewal capacity, demonstrated by their ability to generate neurospheres. Stem cell markers such as Nestin, Melk, Bmi-1, Mush-1, Oct-3/4, and Sox2 were found, as CSC markers like CD133, CD90, SSEA1, and CD44 (20). Cryopreservation was reliable with no loss of the precursor phenotype (77).

Tissue samples were obtained from patients operated at the Neurosurgery Department at Hospital Universitario La Fe (Valencia, Spain). Tissue samples were obtained from adult patients with high-grade GBM who were recruited by the neurosurgery department from “Hospital Universitario la Fe” in Spain and who did not have any infectious contagious disease. Pediatric patients were excluded. Permission to use this material was obtained from the ethical review board in Hospital Universitario La Fe, and fully informed written consents were obtained from the patients who volunteered to participate. The Hospital Universitario La Fe biobank provided the anonymized clinical data. All the studies were conducted after the approval of the Ethics Committee for Clinical Research at HM Hospitales in Spain (Research Ethics Committee code: 17.12.1148-GHM). The patient-derived GSC used in this study has been previously characterized as PN, MES-N, and MES-SN after xenotransplantation into nude mice (20). Briefly, mesenchymal GSCs grow as semi-adherent neurospheres (called GBM38 and GBM18 in previous works); exhibit high glycolytic activity (78); express markers such as CD44, ALDH1A3, and ITGB5; and give rise to tumors more aggressive than PN GSCs. Proneural GSCs (called GBM27) grow as suspension neurospheres, have a high proliferative rate and longer overall survival, and express markers such as CD133, SOX2, miR20b, and miR125b and have a higher expression of OLIG2, consistent with the CSC type I described by Günther et al. (76), and the proneural phenotype (79–82). The selected CSC-enriched cultures grow as 3D neurospheres in serum-free conditions and form tumors when xenotransplanted to immunodeficient mice brain, recapitulating the phenotype and gene expression of the original tumor. The induction of differentiation in these cultures produces the diminution of stem cell markers such as Sox2, CD133, NES, and OLIG2 (83) and gives rise to cells expressing astrocyte, oligodendrocyte, and neuron markers. We found an important number of cells co-expressing several lineage-restricted markers, which have been previously reported and regarded as another feature of the brain tumor (20, 84).

Cell lines were grown as neurosphere cultures in a serum-free DMEM/F-12 (with L-glutamine and 15 mM HEPES) media supplemented with N2, 300 ng/ml hydrocortisone, 2 μg/ml heparin, 30 ng/ml triiodothyronine, 10 ng/ml EGF, 20 ng/ml FGF-2, NEAA 1x, D-glucose, BSA fraction V, sodium pyruvate, L-glutamine, and antibiotic-antimycotic. To minimize the possibility of artifactual results due to changes in the biology of the cells along the passages, all experiments were carried out within 10 passages.

### Enrichment and Isolation of GSCs and Derived EVs

GSCs were cultured in a 225-cm^2^ cell culture flask. Every 3–4 days, the medium was changed with 30 ml fresh media. After 1 week, the medium was changed for the last time and collected after 4 days for EV isolation. For the performance of the UHPLC/Q-TOF-MS experiment, the GSC and the supernatant of ten cell holders (10x 225 flasks) containing the EV from each GSC phenotype were collected.

To avoid chemical contamination as a wreck of the cellular plasmatic membrane, neurospheres were collected together using a pipette and PBS buffer. They were centrifuged at 800 x *g* for 5 min, and the pellet was then washed with PBS and split in different aliquots. A small fraction of the spheres were disaggregated using Accutase^®^ solution (Sigma) to be able to quantify the individual cells with the use of trypan blue in a Neubauer chamber.

The media collected containing the EV were centrifuged at 1,700 x *g* for 10 min to remove cellular debris. Then, this was concentrated using Amicon^®^ Ultra-15 centrifugal filter devices (Millipore) following the manufacturer’s instructions. The concentrated medium was used for the EV isolation through differential and serial ultracentrifugations using an Optima-LE 80K ultracentrifuge (Beckman Coulter) and an SW28 swing rotor. Firstly, the medium was centrifuged at 8,000 x *g* for 20 min to recover the AB. Secondly, centrifugation at 25,000 x *g* for 30 min was performed to obtain MV and the exosomes were subsequently harvested by centrifugation at 100,000 x *g* for 90 min. All centrifugations were performed at 4°C, and all the pellets were washed with PBS at each step and centrifuged again (85). In addition, cell culture medium, not containing cells, was used as blank of the lipidomics experiments. Exosomes were characterized using a NanoSight NS300 instrument (Malvern) and ExoArray. Moreover, previous characterization of EXO, MV, and AB was performed using transmission electron microscopy, dynamic light scattering method, and size distribution with Zetasizer Nano ZS (Malvern Instruments). Full characterization can be found in García-Romero et al. (Oncotarget 2017) (86). Briefly, TEM images revealed the typical morphology and expected diameter ranges of the ABs (>1 μm), MVs (~200 nm), and EXOs (~100 nm). The Zetasizer ranged the fractions as follows: the AB fraction was from 2,000 to 500 nm, the MV fraction showed an average size of 600 nm, and the EXO fraction displayed a main peak at 180 nm.

After isolation, samples were immediately used or stored at −80°C until lipidomics analysis.

### Nanoparticle Tracking Analysis

Nanoparticle tracking analysis (NTA) was performed for exosome quantification and particle size determination using a NanoSight NS500 instrument (Malvern) equipped with a blue laser (405) and the NTA 3.1 analytical software. All experiments were carried out at 1:1,000 dilution in PBS, leading to particle concentrations around 1 × 10^8^ particles/ml. The particle size range was 60–140 nm (**Supplementary Figure 3A**).

### Exosome Array

The identification of protein markers on the isolated exosomes was done using the commercially available Exo-Check exosome antibody array kit (System Biosciences Inc.) as described by the manufacturer. The membrane was developed with SuperSignal West Femto Maximum Sensitivity Substrate (Thermo Fisher Scientific) and analyzed using ChemiDoc (**Supplementary Figure 3B**).

### Lipid Extraction

To prevent metabolite oxidation/degradation, sample treatment and exosome isolation were always performed at a temperature of 4°C, and as soon as the exosomes were isolated, these samples were cooled in liquid nitrogen (87, 88). To ensure ceramide stability, a recent review reported that ceramides can be oxidized only under very forced conditions such as up to 7 days of oxidation where mainly keto, hydroxy, and hydroperoxyl derivatives were observed (87).

Lipid extraction was performed using double extraction with cold methanol (−20°C) (89). For the first extraction, 400 µl of methanol was added for every 10^7^ cells, while 100 µl was added for every 10^15^ particles of exosomes. After methanol addition, samples were vortex-mixed vigorously and ultrasonicated with a bath sonicator for 10 min in cold water. After that, samples went through three cycles of freeze/thaw in liquid nitrogen/cold water for 10 s. Samples were then centrifuged at 4°C at 16,000 × *g* for 20 min (centrifuge 5415 R Eppendorf). The supernatant was transferred to a new tube, and pellets were re-extracted using 200 µl cold methanol for 10^7^ cells and 50 µl for 10^15^ exosomes. The resulting supernatant was collected for each sample and vortexed. For analysis, 45 µl was added into an LC vial containing the same number of cells or exosomes per phenotype. Additionally, individual QC samples for cells and exosomes were prepared independently by pooling and mixing equal volumes of all cells and exosome samples, independently (90).

### UHPLC/Q-TOF-MS Lipidomic Fingerprinting

The lipid profile was determined in a UHPLC consisting of a degasser, a binary pump, and an autosampler (1290 Infinity II, Agilent). A sample volume of 0.5 µl for cells and 1 µl for exosomes were injected to a reversed-phase column (Poroshell 120EC-C8 150 x 2.1 mm, 2.7 µm; Agilent) that was maintained at 60°C during the analysis. The system was operated at a flow rate of 0.5 ml/min with solvent A [positive ionization mode: H_2_O containing 10 mM ammonium formiate; negative ionization mode: H_2_O containing 0.1% of formic acid (FA)] and solvent B [positive ionization mode: methanol:isopropanol (MeOH:i-PrOH; 85:15) containing 0.1%FA]. The gradient was 75% to 96% B (0–23 min), 96% B (23–31 min), 96% to 100% B (31–31.5 min), 100% B (31.5–32.5 min), and 100% to 75%B (32.5–33 min). The system was finally held at 75% B for 7 min to re-equilibrate (total running time of 40 min per analysis). Data were collected in positive and negative ESI modes in separate runs using a mass analyzer of quadrupole time of flight (Q-TOF; Agilent 6550 iFunnel). The analyses were performed in full scan from *m*/*z* 50 to 1,000. The capillary voltage was 3,500 V, and the nozzle voltage was 1,000 V, with a scan rate of 1.0 spectrum per second. The gas temperature was 290°C, the drying gas flow rate was 11 L/min for positive ESI mode and 13 L/min for negative ESI mode, the nebulizer was set to 40 psi, the sheath gas temperature was 350°C, and the sheath gas flow rate was 11 L/min for positive ESI mode and 12 L/min for negative ESI mode. For both modes, the Q-TOF-MS parameters were a fragmentor voltage of 175 V and an octopole radio-frequency voltage of 750 V. During the analyses, two reference masses were used: *m*/*z* 121.0509 [purine, (C_5_H_4_N_4_+H)^+^] and *m*/*z* 922.0098 [HP-0921, (C_18_H_18_O_6_N_3_P_3_F_24_+H)^+^] in positive ESI mode and *m*/*z* 112.9855 [TFANH_4_, (C_2_H_4_O_2_NF_3_-NH_4_)^−^] and *m*/*z* 966.0007 [HP-0921, (C_18_H_18_O_6_N_3_P_3_F_24_+FA-H)^−^] in negative ESI mode. The references were continuously infused into the system, enabling constant mass correction. Samples were analyzed in randomized runs in two continuous independent batches for exosomes and cells, during which they were kept in an autosampler at 4°C. The worklists for both polarities started with the analysis of 10 QC injections for column equilibration; a QC sample was injected at the beginning of the sequence to stabilize the system and throughout the analytical runs at periodic intervals of four samples until the end of the sequence to monitor the stability, performance, and reproducibility of the system.

### Data Treatment

The raw data obtained by the UHPLC/Q-TOF-MS positive ESI mode were cleaned of background noise and unrelated ions by the Batch Recursive Feature Extraction (BRFE) tool included in the MassHunter Profinder B.08.00 software (Agilent). The BRFE algorithm creates a list of masses and retention times, associated with the abundance of the possible components representing the full TOF masses from the spectral data. Each component is the sum of co-eluting ions that are related by charge-state envelope, isotopic distribution, and/or the presence of different adducts and dimmers. In order to find co-eluting adducts of the same feature, the following adducts were selected: +H, +Na, and +NH_4_ for UHPLC/Q-TOF-MS data.

Data were filtered according to these criteria: 1) features found in blanks and 2) absent in more than 50% of QCs. Additionally, data with a coefficient of variation (CV) lower than 30% in the QCs were kept.

### Multivariate Analysis

Multivariate analysis was performed using SIMCA P+14.0 (Umetrics, Umeå, Sweden). PCA, a non-supervised model, was used to observe data patterns using univariate (UV) scaling. The robustness of the models was evaluated based on the R^2^ (explained variance) score.

### Data Analysis and Presentation

Ceramide in cells and exosomes present in each phenotype were calculated by summing up the total area of all ceramide species measured with LC-Q-TOF-MS analysis and then normalizing to the total abundance (%) independently. The final data are presented as mean % with error bars showing mean ± standard error of mean (SEM). The data were analyzed by one- and two-way analyses of the variance (ANOVA) followed by *post-hoc* multiple comparison Tukey range test as indicated accordingly. In all cases, *, p < 0.05; **, p < 0.01; and ***, p < 0.001. For all bar graphs, error bars represent the mean ± SEM.

### Workflow for Ceramide Annotation

The annotation process was carried out in two steps: the first one was an initial tentative identification of the statistical significance features, based on the MS1 data, using CEU Mass Mediator (CMM) (91, 92). This stage started with the tentative assignment of the experimental accurate masses, tolerance 5 ppm error, with the candidate hits retrieved from the database, covering retention time and isotopic distribution including possible ions and adducts.

Secondly, to increase the level of confidence ceramide annotation, samples were analyzed in negative and positive electrospray ionization mode using the iterative MS/MS mode function with fixed collision energy at 20 and 40 eV. Iterative MS/MS data were searched in the Agilent Lipid Annotator software tool (version 1.0) (93). Then, targeted MS/MS data were analyzed manually in the Agilent MassHunter software (version 8.0) using retention time and MS/MS fragmentation matching.

We also explored the fragmentation pattern of MS/MS spectra of authentic standards available and the predicted MS/MS spectra in resources at LIPID MAPS (www.lipidmaps.org) for the corresponding ceramides in our study.

### Nomenclature

The designations and abbreviations used follow the updated shorthand nomenclature for description of ceramides at different levels for characterization (94) as observed in **Supplementary Figure 4**.

## Data Availability Statement

The original contributions presented in the study are included in the article/**Supplementary Material**. Further inquiries can be directed to the corresponding authors on reasonable request.

## Ethics Statement

The studies involving human participants were reviewed and approved by the Ethics Committee for Clinical Research at HM Hospitales in Spain (Research Ethics Committee code; 17.12.1148-GHM). The patients/participants provided their written informed consent to participate in this study.

## Author Contributions

Conceptualization, CB and AA-S. Methodology, RM-FdM and DR. Formal analysis, RM-FdM, AV, and AG. Investigation, RM-FdM, AV, DR, AG and JC-N. Writing, original draft preparation, RM-FdM, AV, and AG. Writing, review, and editing, CB, DR, and AA-S. Supervision, CB, AA-S, DR, AV, and AG. All authors contributed to the article and approved the submitted version.

## Funding

This work and RM-FdM were funded by a Marie Curie grant: Applying Metabolomics to Unveil follow-up treatment biomarkers and Identify Novel Therapeutic Targets in Glioblastoma (MaGMa), number 799378. This work was also supported by the grant from Ministerio de Ciencia, Innovación y Universidades-FEDER RTI2018-095166-B-I00, the “Fondo de Investigaciones Sanitarias” (FIS) (PI17-01489), del Instituto de Salud Carlos III (AA-S), and the Ministerio de Economía y Competitividad–FEDER (RTC-2016-4990-1) (AA-S). AV is funded by a postdoctoral research fellowship from ARADyAL.

## Conflict of Interest

The authors declare that the research was conducted in the absence of any commercial or financial relationships that could be construed as a potential conflict of interest.

## Publisher’s Note

All claims expressed in this article are solely those of the authors and do not necessarily represent those of their affiliated organizations, or those of the publisher, the editors and the reviewers. Any product that may be evaluated in this article, or claim that may be made by its manufacturer, is not guaranteed or endorsed by the publisher.
