# Supplementary material for: Ceramide Composition in Exosomes for Characterization of Glioblastoma Stem-Like Cell Phenotypes
Source: Front Oncol. 2022 Jan 21;11:788100. doi: 10.3389/fonc.2021.788100 (PMC8814423; doi:10.3389/fonc.2021.788100)
Supplement: Supplementary file 1 [file DataSheet_1.docx]

Supplementary Material


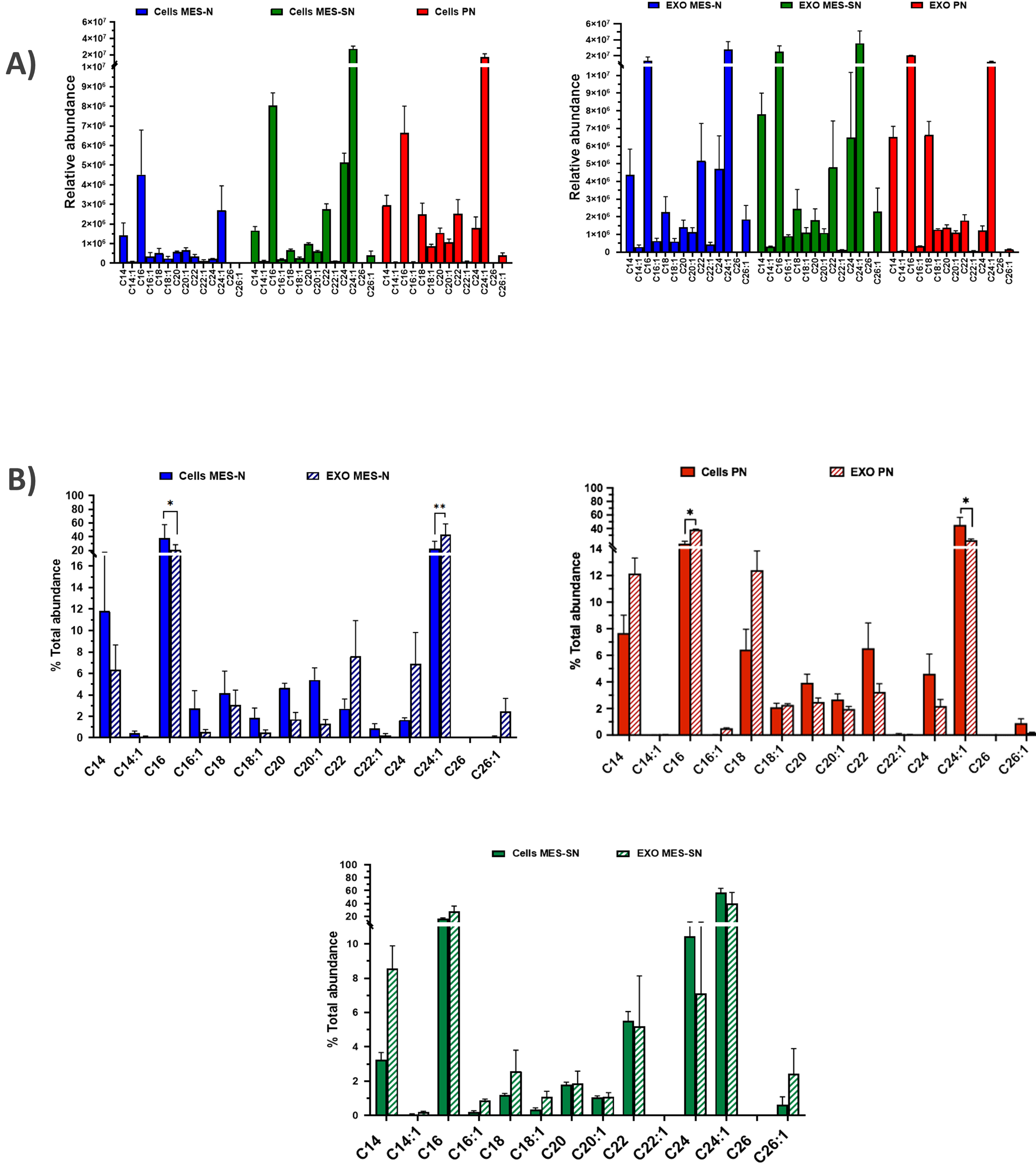


**Supplementary Figure 1.** **Zoomed graphs of the ceramide profiles abundance.** A) Ceramide profile for the three phenotypes for cells and EXO. B) Ceramide distribution between EXO and their parent cells for each phenotype independently. In blue: MES-N phenotype; in green: MES-SN phenotype and in red PN phenotype.

|  | **MES-SN** | **MES-N** | **PN** |
| --- | --- | --- | --- |
| **CSCs** N**EUROSPHERES** | 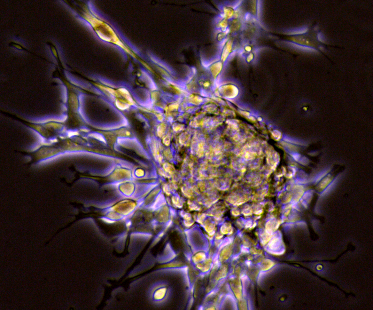 | 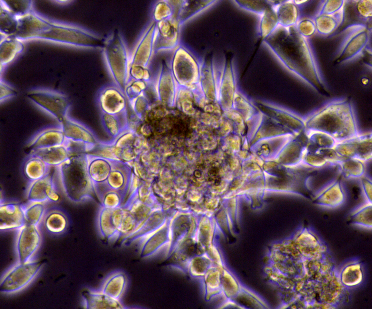 | 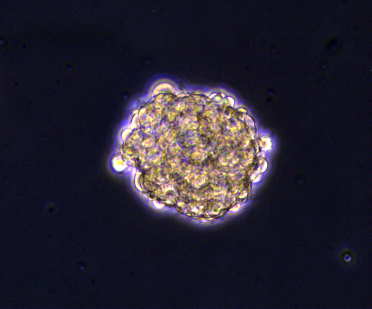 |

**Supplementary Figure 2: Characterization of GSCs.** The GSCs cultures were generated and maintained as neurosphere cultures in a serum-free selective for CSCs described in methods. Light microscopy images of neurospheres (GSCs) obtained from primary cultures.


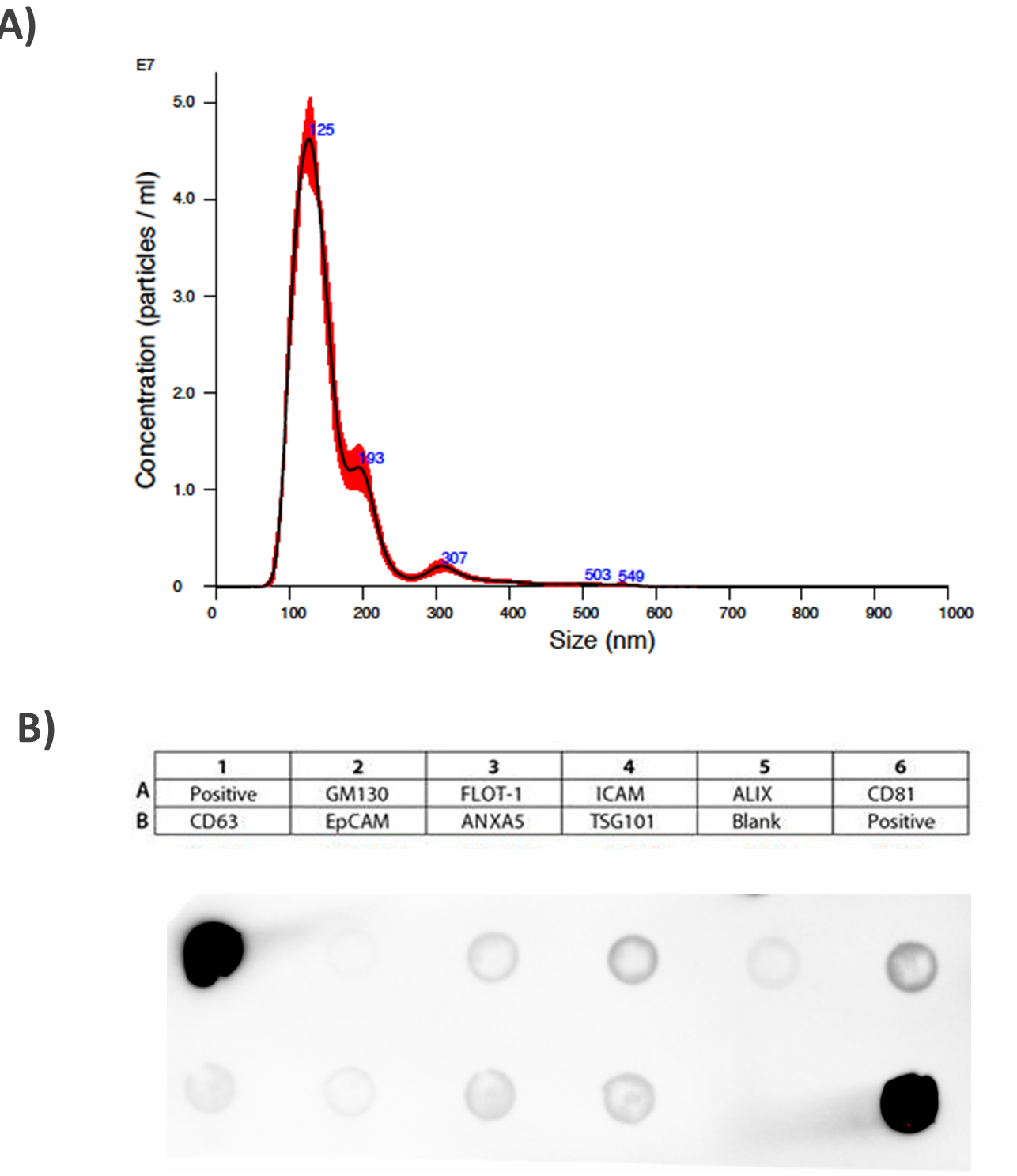


**Supplementary Figure 3**. **Exosome characterization using Nanoparticle tracking analysis (NTA) and Exo-Check Exosome antibody Array**. A) All the exosome samples were quantify using NTA by triplicate. One representative experiment is shown. B) Representative image of one Exosome Array assay out of 3 replicates. A1 and B6: positive control for HRP detection, A2: GM130 Cis-golgi matrix protein, A3: FLOT1, Flotillin-1, A4: ICAM1 Intercellular adhesion molecule 1, A5: ALIX, Programmed cell death 6 interacting protein (PDCD6IP), A6: CD81, Tetraspanin, B1: CD63 Tetraspanin, B2: EpCam Epithelial cell adhesion molecule, B3: ANXA5 Annexin A5, B4: TSG101 Tumor susceptibility gene 101, B5: Blank Background control.


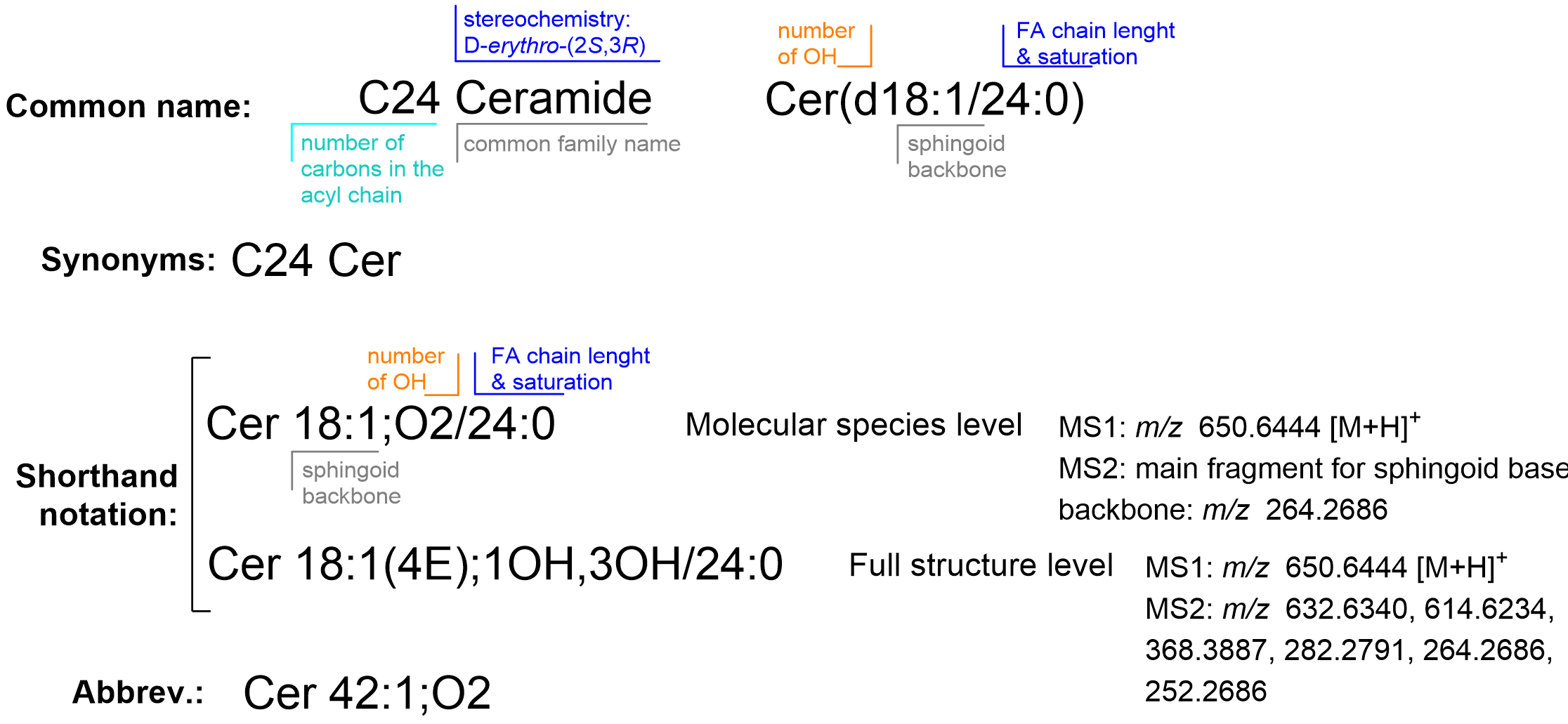


**Supplementary Figure 4. Ceramide designations and abbreviations used for annotation.**
